# Evaluation of Chikungunya Diagnostic Assays: Differences in Sensitivity of Serology Assays in Two Independent Outbreaks

**DOI:** 10.1371/journal.pntd.0000753

**Published:** 2010-07-20

**Authors:** Grace Yap, Kwoon-Yong Pok, Yee-Ling Lai, Hapuarachchige-Chanditha Hapuarachchi, Angela Chow, Yee-Sin Leo, Li-Kiang Tan, Lee-Ching Ng

**Affiliations:** 1 Environmental Health Institute, National Environment Agency, Singapore, Singapore; 2 Communicable Disease Centre, Tan Tock Seng Hospital, Singapore, Singapore; Centers for Disease Control and Prevention, United States of America

## Abstract

**Background:**

The sensitivity and specificity of two in-house MAC-ELISA assays were tested and compared with the performance of commercially-available CTK lateral flow rapid test and EUROIMMUN IFA assays for the detection of anti-Chikungunya virus (CHIKV) IgM. Each MAC-ELISA assay used a whole virus-based antigen derived from genetically distinct CHIKV strains involved in two chikungunya disease outbreaks in Singapore (2008); a January outbreak strain with alanine at amino acid residue 226 of the E1 glycoprotein (CHIKV-A226) and a May-to-September outbreak strain that possessed valine at the same residue (CHIKV-226V). We report differences in IgM detection efficacy of different assays between the two outbreaks. The sensitivities of two PCR protocols were also tested.

**Methods and Findings:**

For sera from January outbreak, the average detection threshold of CTK lateral flow test, MAC-ELISAs and EUROIMMUN IFA assays was 3.75, 4.38 and 4.88 days post fever onset respectively. In contrast, IgM detection using CTK lateral flow test was delayed to more than 7 days after fever onset in the second outbreak sera. However, MAC-ELISA using CHIKV-226V detected IgM in the second outbreak sera 3.96 days after fever onset, which was approximately one day earlier compared to the same assay using CHIKV-A226 (4.86 days). Specificity was 100% for both commercial assays, and 95.6% for the in-house MAC-ELISAs. For sensitivity determination of the PCR protocols, the probe-based real time RT-PCR method was found to be 10 times more sensitive than one based on SYBR Green.

**Conclusion:**

Our findings suggested that the two strains of CHIKV using variants A226 and 226V resulted in variation in sensitivities of the assays evaluated. We postulated that the observed difference in antigen efficacy could be due to the amino acid substitution differences in viral E1 and E2 envelope proteins, especially the E1-A226V substitution. This evaluation demonstrates the importance of appraisal of different diagnostic assays before their application in clinical and operational settings.

## Author Summary

Chikungunya is a mounting public health concern in many parts of the world. Definitive diagnosis is critical in differentiating the diseases, especially in dengue endemic areas. There are some commercial chikungunya kits and published molecular protocols available, but no comprehensive comparative evaluation of them was performed. Using sera collected in outbreaks caused by two variants of Chikungunya virus (A226 and 226V), we tested 2 commercial IgM tests (CTK lateral flow rapid test and EUROIMMUN IFA) alongside our in-house IgM assays (using both variants of the virus). Sensitivities of 2 published PCR protocols were also evaluated based on RNA standards derived from cell-cultured viruses. The commercial assays had different performances in each outbreak, with CTK's lateral flow test having the best performance in the first outbreak and EUROIMMUN IFA being more sensitive in the second outbreak. Use of the current circulating virus in a test assay improves sensitivity of the MAC-ELISAs. For PCR, a probe-based real time RT-PCR method was found to be 10 times more sensitive than the SYBR Green method. Despite this, the latter protocol is found to be more suitable and cost-effective for our diagnostic laboratory. This evaluation demonstrates the importance of appraisal of commercial kits and published protocols before application of a diagnostic tool in the clinical and operational setting.

## Introduction

Chikungunya virus (CHIKV) has seen a resurgence in recent years, with outbreaks being described in Republic of Congo in 2000, La R'eunion in 2005, India, Sri Lanka, Malaysia and Gabon in 2006, Italy in 2007, Singapore and Thailand in 2008 [1], [2], [3], [4], [5], [6], [7], [8], [9]. The current pandemic involves a newer CHIKV strain of the East-Central South African (ECSA) genotype. Extensive research and analysis demonstrated the role of a viral mutation, A226V, in the changed epidemiology of the disease. There is no evidence that this particular mutation caused any alteration in virulence of the CHIKV or clinical manifestations of the disease, but the mutation, residing in the viral envelop protein, has been shown to facilitate enhanced transmissibility of the virus by *Aedes* (*Ae.*) *albopictus*. Several sophisticated studies have established that the A226V mutation rendered higher viral replication and dissemination rates in *Ae. albopictus*, and thus shortening the extrinsic incubation period in the vector [10], [11]. The length of extrinsic incubation period determines the infective life span of a vector, and consequently has great influence on the epidemic potential of the virus-vector partnership.

Since 2006, in response to the outbreaks in the region, the Environmental Health Institute (EHI), a national public health laboratory in Singapore, has initiated laboratory surveillance for CHIKV. Two main outbreaks were detected: the first occurring in January 2008 was a small outbreak with 13 local cases [12], [13]; and the second commenced in May 2008 and peaked two months later, resulting in 231 local cases by the end of September 2009 [13].

Phylogenetic analysis concluded that viruses isolated from these two outbreaks were related to the ECSA genotype [13]. Interestingly, the viruses from the first outbreak showed alanine at amino acid residue 226 (A226) of *E1* gene and those from the second outbreak showed valine (226V) at the same codon. While *Ae. aegypti* was the implicated vector in the first outbreak, *Ae. albopictus* was the confirmed vector of the second outbreak [13]. Though CHIKV was not isolated from any field caught *Ae. aegypti* during the first outbreak, entomological investigations in the affected area found only *Ae. aegypti* adults, and data from routine surveillance (part of Singapore dengue control programme) also showed that *Ae. aegypti* was the predominant species in the area. On the other hand, CHIKV was isolated from *Ae. albopictus* caught during the second outbreak.

Laboratory confirmation of CHIKV infection is critical, especially in dengue endemic areas, as clinical symptoms of the two diseases are similar. However the two viruses may be transmitted by different vectors (*Ae. aegypti* and *Ae. albopictus*), which require different control strategies. RT-PCR is an excellent tool for the early phase confirmation of CHIKV infections, and many protocols have been established for this purpose [13], [14], [15], [16], [17], [18], [19], [20], [21]. Unfortunately, this viral detection method is limited to the viraemic phase, which is usually one to five days after fever onset. Thereafter, confirmation of CHIKV infection requires serological tests. In recent years, a few commercial CHIKV diagnostic kits have emerged in the market, but there are very few reports on the systematic and comparative evaluation of these commercial products. The most recent CHIKV diagnostic assay on the market is the indirect immunofluorescence assay (IFA) from EUROIMMUN AG (Lübeck, Germany), whose IgM assay presented a specificity of 98.3% and a sensitivity of 96.9% [22]. This assay, together with an IgM lateral flow rapid test kit by CTK Biotech Inc (San Diego, USA), was evaluated alongside an in-house IgM Capture Enzyme-Linked Immunosorbant Assay (MAC-ELISA). Sensitivities, specificities and approximate time antibodies first became detectable in an infected patient were determined. A comparison between the sensitivity of two PCR protocols was also performed using RNA standards derived from cell-cultured viruses. The evaluation, using samples from two independent and epidemiologically distinct CHIKV outbreaks in Singapore, was done to establish diagnostic capability in the laboratory. The IgM titres were not determined, and thus the kinetics of the antibody has not been established. However, the availability of a reliable assay allows antibodies to be titred for each sample, and thus facilitating an ongoing antibody kinetics study, that will be reported later.

## Materials and Methods

### Data and Sample Collection

Environmental Health Institute is a public health laboratory that functions as a licensed diagnostic laboratory, with an ISO9001 accreditation. It served as the national reference laboratory during the CHIKV outbreaks in 2008. The three diagnostic techniques were evaluated using three characterized panels of sera.

Persons with an acute febrile illness, signs or symptoms compatible with chikungunya fever (fever, joint pain, or rash) were tested with CHIKV RT-PCR [12], a routine test which has been offered under EHI's quality assured programme as required for the national license. Sera panel A and B were multiple consecutive samples collected from RT-PCR CHIKV confirmed patients, during the first and second CHIKV outbreaks respectively (See supporting information “Supporting Data S1”). The patients were warded at the Tan Tock Seng Hospital Communicable Disease Centre (TTSH CDC). The first samples were collected on the day of first medical consultation and subsequently, more samples were collected as the disease progressed, till convalescence. The daily samples were used to determine the sensitivity of IgM serology on each day of illness.

Panel A, comprising residual blood from eight CHIKV-confirmed patients from the first outbreak (January 2008), were collected for clinical management and to determine when the patient could be discharged. Six to 11 samples were collected from each patient, resulting in a total of 60 samples (See supporting information “Supporting Figure S1”).

Panel B, from the second outbreak (May to September 2008) was collected from 28 CHIKV-confirmed patients in August 2008 prospectively. Each patient had five to 12 samples collected, leading to a total of 225 samples.

All sera samples were kept at 4°C after phlebotomy, transported on ice, and reached the laboratory within 24 hours. The samples were either tested on the same day, or stored at −80°C until testing.

Panel C sera were used for specificity tests and consisted of 45 flavivirus-confirmed sera (44 Dengue, one Japanese Encephalitis) and five non-CHIKV alphavirus-confirmed sera (two Barmah Forest, three Ross River).

Analysts of the serology tests were not blinded to the RT-PCR results of the first samples. However, they were blinded to the serology results derived from the other tests.

### Ethics Statement

Panels A and C were residual samples of sera sent to EHI for diagnosis. Use of residual samples for evaluation of diagnostic assays to establish in-house capability is exempted from internal review by the National Environment Agency Bioethics Review Committee. Use of sera panel B, which was collected for a larger study, was approved by the National Healthcare Group Domain Specific Review Board, and written informed consent was obtained from the study participants.

### Serological Assays

#### In-house IgM capture Enzyme-Linked Immunosorbant Assay (MAC-ELISA)

Native, inactivated antigens of CHIKV D67Y08 [isolate name SG(EHI)chik672008] and D1225Y08 [isolate name SG(EHI)CHD122508] isolates from patients were used for the in-house MAC-ELISA. The former had alanine at amino acid residue 226 (A226) of *E1* gene and the latter, isolated in the second outbreak, had valine (226V) at the same codon. CHIKV D67Y08 (A226) and D1225Y08 (226V) were passaged twice through Vero cells (ATCC No. CCL-81) before their supernatents were collected and virus titres were determined using a plaque assay method as previously described [23]. CHIKV antigens were heat inactivated before use. Supernatant of each CHIKV strain was diluted with Casein buffer to attain a titre of 10^6^ pfu/ml. A single batch of antigen was prepared from each virus. The same batches of antigens, controls and reagents were utilized for the entire evaluation. Testing with reference CHIKV IgM positive serum obtained from the Royal College of Pathologist of Australasia Quality Assurance Programme (RCPA QAP), yielded equivalent optical density (OD) values for the two assays with different viruses, and verified the standardization performed. However, no calibration with any sera was performed as that would have eliminated any differences observed for different sera. The MAC-ELISA was adapted from Taiwan Communicable Disease Centre, Taipei [personal comm]. All reagents were added at 100 µl per well. Briefly, 96-well maxisorp plates (Nunc, Denmark) were coated overnight at 4°C or one hr at 37°C with 2.6 µg/ml of goat anti-human IgM Fc_5u_ (Jackson ImmunoResearch, USA) in sodium bicarbonate (pH 9.5). Wells were then washed once with washing buffer (PBS and 0.05% Tween-20) and blocked in dilution buffer (Casein buffer and 0.05% Tween-20) for one hr at 37°C. Wells were subsequently washed once and 1∶100 diluted sera were added. In each plate, two positive controls, two negative (Dengue IgM human sera) and one plate control (no sera added) were included. The two positive controls (CHIKV IgM human sera with a titre of 1∶256) were derived from each outbreak. After incubation for one hr at 37°C, the wells were washed six times. CHIKV antigen (10^6^ pfu/ml) was added into each well and incubated for two hrs at 37°C. After washing, 1∶50 mouse anti-alphavirus IgG (Santa Cruz Biotechnology, USA) was added into each well and incubated for two hrs at 37°C. After subsequent washing, 1∶1000 dilution of peroxidase conjugated rabbit anti-mouse IgG (DAKO, Denmark) was added into each well and incubated for one hr at 37°C. Tetramethylbenzidine (TMB) substrate (Sigma, USA) was added and reaction was stopped with 1N sulphuric acid. Absorbance was measured at 450 nm against a reference filter at 620 nm.

Cut-off values were derived by using mean ± standard deviation OD values of the negative controls. A sample was considered negative when the OD value was less than the mean value of the negative control plus two standard deviations, considered equivocal when the OD value exceeded the mean value of the negative control plus two standard deviations, but less than three standard deviations, and considered positive when the OD value is above three standard deviations. In this study, all equivocal results were retested.

#### Commercial kits

Each batch of commercial assays was checked with a CHIKV IgM positive reference serum obtained from the RCPA QAP, before usage. Chikungunya IgM IFA from EUROIMMUN AG (Lübeck, Germany) (catalog no. FI 293a-1010M) and On-site Chikungunya IgM lateral flow rapid test from CTK Biotech Inc (San Diego, USA) (catalog no. R0065c) were utilized according to manufacturers' instructions. In brief, for EUROIMMUN IFA, CHIKV IgM serum samples were diluted 1∶10 with the diluent buffer provided, and 25 µl of this was used for each reaction. Eight samples could be run on each slide, alongside one positive and one negative control. Slides were incubated for 30 mins at room temperature before washing with PBS-Tween buffer provided. Then, 20 µl of Fluorescein-labelled anti-human IgM was added and this was followed by a dark incubation at room temperature for another 30 mins, before slides were mounted and visualized under the fluorescence microscope. A positive reaction occurred when cells fluoresced. For CTK rapid cassette, 35 µl of a serum sample and a drop of the provided sample diluent were applied onto the sample well. Each cassette was incubated at room temperature for 10 mins. A positive reaction was when the test line appeared alongside the control line.

EUROIMMUN IFA uses the whole virus of CHIKV-A226 [22], [24] whereas CTK uses a recombinant antigen that included alanine at amino acid residue 226 of *E1* gene (CHIKV-A226) [personal comm].

### RT-PCR

To determine the sensitivities of PCR protocols, two previously published protocols were tested. The first was a one-step SYBR Green based RT-PCR, from Hasebe *et al.* 2002, for the detection of a fragment of the non-structural protein 1 (nsP1) gene of CHIKV [15]. The PCR conditions were described in Hapuarachchi *et al.* 2010. The other assay was a taqman probe-based RT-PCR protocol adapted from Pastorino *et al.* 2005, with slight modifications to the primers which target the *E1* region (Table 1) and included following modifications to the PCR assay: PCR assay was performed using the Qiagen QuantiTect Probe RT-PCR kit, in a final reaction volume of 20 µl containing 5 µl of template, 1× of buffer mix, 0.2 µl of RT enzyme mix, 0.25 µM of probe, 0.25 µM and 0.4 µM of forward and reverse primers respectively. The amplification cycles were extended to 50 with denaturation at 94°C for 5 sec and annealing/extension step at 60°C for 1 min. The analytical sensitivity and reproducibility of both assays were determined using 10-fold dilutions of cultured CHIKV strains D67Y08 (A226) and D1225Y08 (226V) (2.7×10^−1^ to 2.7×10^8^ pfu/ml). CHIKV RNA was extracted from the dilutions using QIAamp viral RNA mini kit (Qiagen, Hilden, Germany).

**Table 1:** Primers and probe sequences of Taqman probe-based RT-PCR assay.

Name	Sequence (5′→3′)	Position (based on full genome)	Position based on complete *E1* gene
Forward Primer (10366F)	AAG CTY CGC GTC CTT TAC CAA GGA AA	10366 to 10391	(9952 to 11268bp)
Reverse Primer (10574R)	CCA AAT TGT CCT GGT CTT CCT	10574 to 10554	393 to 418
Taqman Probe (P10465)	(Fam)CCA ATG TCY TCM GCC TGG ACA CCT TT(TAMRA)	10465 to 10490	581 to 601

### Test and Data Analysis

A total of three analysts were involved in the evaluations. The two commercial IgM assays were performed by one analyst, and the in-house ELISA assays were performed by another. The third analyst performed the PCR sensitivity tests. All analysts were trained in-house, certified by the Director of the diagnostic laboratory, and regularly passed the external (RCPA) and internal proficiency tests, under the EHI quality assurance programme.

The sensitivity and specificity of assays were calculated in Microsoft Excel 2007. ANOVA, to test for variance amongst results obtained by the serological assays, and Student t-tests to determine any significant differences between the different assays were calculated using SPSS 13.0 software.

## Results

### Average Detection Threshold, Sensitivity and Specificity of Serological Assays

The commercial lateral flow rapid test and IFA, along with in-house MAC-ELISAs using both D67Y08 (226A) and D1225Y08 (226V), were evaluated using three panels of sera. Sera panels A and B were collected during the two outbreaks in January and May to September in 2008 respectively, and were from CHIKV RT-PCR-confirmed patients (See supporting information “Supporting Data S1”). Average IgM detection threshold, according to day after fever onset was determined. During the first outbreak, the lateral flow (CTK) kit enabled the detection of IgM on an average of 3.75 days after fever onset. IFA (EUROIMMUN) and in-house MAC-ELISA detected IgM on 4.88 day and 4.38 day respectively. The use of CHIKV-A226 or -226V did not alter the effectiveness of the in-house ELISA assays (Table 2) during the January outbreak.

**Table 2:** Average detection threshold: Day after onset of fever when IgM was detectable by the evaluated techniques.

	Average day (range of days) of IgM detection after fever onset			
	Lateral flow (CTK)	IFA (EUROIMMUN)	MAC-ELISA (A226)	MAC-ELISA (226V)
**Panel A- January Outbreak (n = 60)**	3.75^a^ (1–7)	4.88^a^(3–6)	4.38^a^(2–5)	4.38^a^(2–5)
**Panel B- May–September Outbreak (n = 225** * **)**	>7* (9–40)	4.86^b^^,^ c(2–7)	4.86^b^^,^ d(2–7)	3.96^b^^,^ c ^,^ d(2–6)
**Overall**	ND	4.86	4.75	4.06

The day of IgM detection was calculated based on sequential samples collected from eight patients in the first outbreak (n = 60), and from 28 patients in the second outbreak (n = 225), and is the mean of the days in which IgM was first picked up by the various assays. For evaluation of CTK assay in the second outbreak, only samples from the first ten patients were used (n = 74).

a Variance between assays, as calculated by ANOVA, demonstrated no significant difference p = 0.56.

b Variance between assays, as calculated by ANOVA, demonstrated significant difference p = 0.003 (CTK lateral flow was disregarded in the analysis).

c T-test showed significant difference between IFA(EUROIMMUN) and MAC-ELISA (226V) p<0.0001.

d T-test showed significant difference between MAC-ELISAs (226V) and (A226) (p<0.0001.

*: Only 10 patients and their subsequent samples were tested.

Though CTK's lateral flow assay had the best performance in the January outbreak, its performance was not repeated in the second outbreak. Among the first 10 CHIKV- confirmed patient sera (total of 74 samples from Panel B), none had detectable IgM within seven days after fever onset. The earliest IgM detection attained by the lateral flow assay, was day nine after the onset of fever (n = 1) and eight of the 10 patients did not show seroconversion even after 14 days. To investigate if the drop in performance was due to batch variability of the CTK kit, 30 CHIKV-A226 IgM positive and 10 CHIKV negative samples from the first outbreak were retested with the second batch of CTK kits. No difference in results interpretation was observed. As the ineffectiveness of the kit was clearly demonstrated by the 74 samples from the 10 patients, and both batches of kits were no longer available, the rest of the samples from the second outbreak (151 samples from 18 patients) were not tested with the CTK assay.

Using IFA (EUROIMMUN) and MAC-ELISA (A226) on the panel collected from the second outbreak, the average day of IgM detection was 4.86 after fever onset. Interestingly, the use of CHIKV-226V as antigen in MAC-ELISA increased the sensitivity to 3.96 day after onset of fever (p<0.0001).

**Figure 1:**
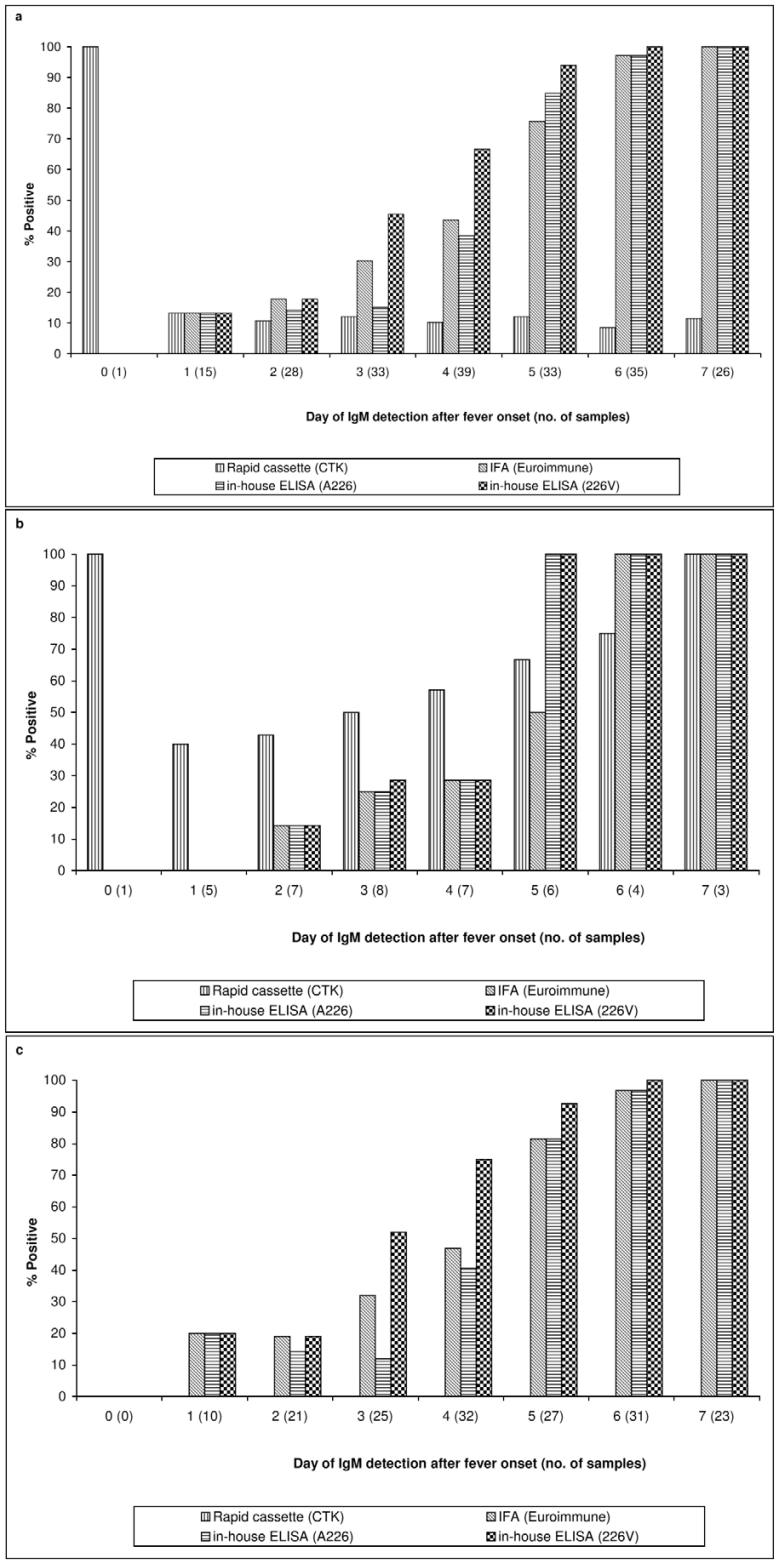
Longitudinal variation of the sensitivity of IgM assays in the first seven days after fever onset. 1a: overall sensitivity based on samples collected from first and second chikungunya fever outbreaks in January and May to September 2008, respectively; 1b: sensitivity based on samples collected from the first outbreak due to CHIKV-A226 strain; 1c: sensitivity based on samples collected from the second outbreak due to CHIKV-226V strain. The numbers in brackets along the x-axis represent the number of samples tested for each day after onset of fever.

Overall, the sensitivity of assays increased along with the progression of the disease (Figure 1). Sensitivities of all assays were very low from day zero to day four of the disease, ranging from 0 to 66.7% (Figure 1a). Sensitivity improved from day five, when MAC-ELISA (226V) fared the best at 93.94%; followed by MAC-ELISA (A226) at 84.85%; and IFA (EUROIMMUN) at 75.76%. Lateral flow (CTK) remained insensitive at 12.12%. By the sixth day, 100% sensitivity was attained by MAC-ELISA (226V), and by day seven, MAC-ELISA (A226) and IFA (EUROIMMUN) also achieved 100%.

In view of the possible variation in sensitivity between the two outbreaks, the samples from the CHIKV-A226 and CHIK-226V outbreaks were analysed separately (Figure 1b). In the first outbreak involving CHIKV-A226, lateral flow (CTK) test was positive in two out of five samples that were collected one day after fever onset. The sensitivity steadily increased to 100% by seven days. The other three assays started to register sensitivities greater than 50% on day five after fever onset.

In the second outbreak, involving CHIKV-226V, MAC-ELISA (226V) had the highest sensitivity of 75% at four days after fever onset and increased to 100% by day six (Figure 1c). IFA (EUROIMMUN) and MAC-ELISA (A226) detected less than 50% of samples on day four, and attained 100% only on day seven. It appeared that in the second outbreak, MAC-ELISA (226V), which utilized the virus isolated in the same outbreak, was the most sensitive.

Panel C comprising of non-CHIKV sera was utilized to determine the specificity that turned out to be 100% for both commercial assays. The sensitivity of in-house MAC-ELISA was 95.6%. The latter picked up two dengue confirmed sera which were paired samples from a single patient collected 14 days apart. Though MAC-ELISA yielded positive results with the two samples, no increase in titre was observed and negative CHIKV PRNT results were obtained (data not shown).

### Determination of Sensitivity of PCR Assays

**Figure 2:**
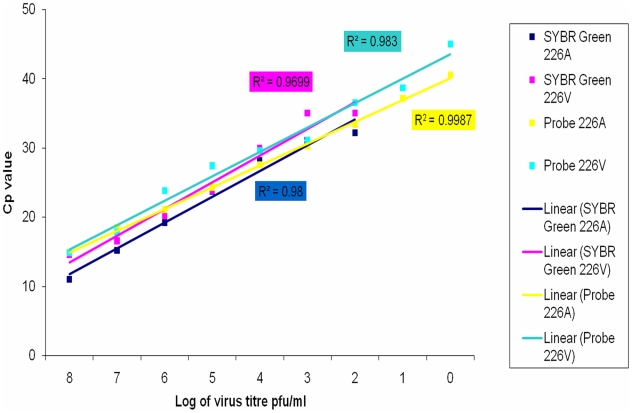
Inverse relationship of Cp values with virus titre when 2 PCR protocols using CHIKV-A226 and -226V were evaluated. Both assays, when tested on CHIKV-A226 and -226V, indicate excellent linear reliability between the decrease of Cp values and the increase in virus titre.

Two real-time PCR protocols were evaluated on RNAs extracted from a serial dilution of each of the cell-cultured viruses, D67Y08 (A226) and D1225Y08 (226V), isolated from the two outbreaks. As relative sensitivity of PCR protocols can be validated using RNA from isolated virus, no patient samples were used for this purpose. The sensitivities of each protocol for both viruses were equivalent. However, the sensitivity of the taqman probe-based protocol was found to be 10 times (2.17×10^0^ pfu/ml) more than the SYBR Green assay (2.17×10^1^ pfu/ml) (Table 3). The inverse relationship of Cp values and virus titres showed very good linear correlation between the detection of CHIKV-A226 and -226V when either assays were used (Figure 2).

**Table 3:** Sensitivity of each CHIKV RT-PCR assay: SYBR Green and Taqman probe-based on PFU per reaction and PFU/ml of cell-culture supernatants of CHIKV D67Y08 and D1225Y08.

Pfu/reaction	Pfu/ml	CHIKV-A226		CHIKV-226V	
		SYBR Green (Cp value)	Taqman probe based (Cp value)	SYBR Green (Cp value)	Taqman probe based (Cp value)
10^5^	2.17×10^7^	11.01	14.94	14.57	14.87
10^4^	2.17×10^6^	15.17	18.47	16.57	17.89
1000	2.17×10^5^	19.17	21.11	20.21	23.82
100	2.17×10^4^	23.78	24.29	23.84	27.45
10	2.17×10^3^	28.16	27.55	29.94	29.60
1	2.17×10^2^	31.15	30.25	>35	31.11
0.1	2.17×10^1^	**32.18**	33.36	**>35**	36.55
0.01	2.17×10^0^	Not detectable	37.15	Not detectable	38.68
0.001	2.17×10^−1^	Not detectable	**40.51**	Not detectable	**>45**

Amplification graphs were checked for cross-point (Cp) value of the PCR product. Cp represented the cycle by which the fluorescence of a sample increased to a level higher than the background fluorescence in the amplification cycle. Melting curve analysis was performed after amplification with the SYBR Green protocol, to ensure that the right product was amplified. No differences in sensitivies were observed when both PCR protocols were tested on either virus. Melting peak was not detected in the SYBR Green assay when less than 0.1 pfu of virus was used in each reaction. However, the probe-based protocol could detect up to 0.01 pfu per reaction. Cp value above 40 is not reliable, and is thus reported as equivocal.

## Discussion

As dengue and chikungunya infections elicit similar symptoms and can be present in the same locations, clinical differentiation may be difficult. In Singapore, it was found that the major chikungunya outbreak in the second half of 2008 was transmitted by *Ae. albopictus*, an outdoor mosquito. Control of chikungunya fever was thus different from the strategy employed for control of dengue fever, which is transmitted by *Ae. aegypti*, a peri-domesticated mosquito. It is thus important to ascertain the cause of a cluster of febrile illness. This study was carried out to ensure that accurate and robust diagnostic tools were used to diagnose chikungunya fever in Singapore.

Our findings suggested that two variants of CHIKV, A226 and 226V, had resulted in variation in sensitivities of the assays evaluated. Though CTK's lateral flow rapid test was found to be a reliable kit in the first outbreak (January 2008), it was ineffective in the second ((May to September 2008). Retesting a panel of CHIKV characterized samples from the first outbreak showed that the inconsistency was not a result of batch variation. We postulated that the inconsistency may be due to the different CHIKV variants involved in two outbreaks. The CTK rapid test kit used a recombinant antigen covering the 226 residue of *E1* gene derived from the CHIKV-A226 [personal comm.], which was similar to the strain involved in the first outbreak. It is thus suggested that CHIKV-A226 derived recombinant antigen was specific for recognition by antibodies elicited by the A226 virus circulating during the first outbreak, but not for those elicited against the 226V virus of the second outbreak. Therefore, it is highly likely that the sensitivity of the rapid test can be improved by including the recombinant antigen derived from the variant virus.

The variation in sensitivity of an assay due to different antigens used was also demonstrated by our in-house MAC-ELISA, where CHIKV-226V antigens yielded higher sensitivity than CHIKV-A226, when tested on sera obtained from patients infected with the CHIKV-226V strain. Similarly, the decrease in sensitivity of the EUROIMMUN IFA was probably attributed to the use of CHIKV-A226 [22], [24]. The less striking sensitivity differences in these assays may be attributed to the use of whole viruses that offer more epitopes for recognition. Interestingly, both A226 and 226V viral strains offered the same sensitivity among samples collected from the first outbreak (A226). Notwithstanding the latter observation, our results indicated that sensitivity of a test could be improved by using the circulating virus isolated during a particular outbreak. It is unlikely that the difference in sensitivity was due to differences in quality of the antigen, as a single batch of antigen was prepared from each virus and yet, sera from the two outbreaks were giving different results for the A226 antigen (but not for the 226V antigen). Both antigens were prepared in the same way, and all sera were tested in one experiment using the same reagents and controls. Our results suggest the disadvantage of using a recombinant antigen that is too specific.

The commercial assays displayed excellent specificity, but the in-house ELISAs picked up two paired dengue IgM samples. These samples did not demonstrate increase in CHIKV IgM titres, and were CHIKV PRNT negative. As such, these were not Dengue and Chikungunya co-infected samples, rather a false positive due to non-specific IgM reactions. In our experience with dengue diagnosis, this phenomenon is not unknown, especially among adults who suffer from conditions such as Systemic Lupus Erythematosus or other immunological conditions. Though the phenomenon is poorly understood, we believe that false positives in IgM serology could be due to other immunological factors, and this may also be the case for CHIKV infection. The cross reaction may not be due to Dengue virus cross- reacting with the CHIKV assay. Our investigation and analysis in Singapore using geographical information system had also revealed that Singapore's major outbreak, due to CHIKV-226V and *Aedes albopictus*, did not overlap spatially with dengue fever, which is transmitted by *Ae. aegypti*. The likelihood of co-infection in Singapore was thus assessed to be very low.

A comparison of the amino acid sequences of the non-structural and structural polyproteins of D67Y08 (A226) and D1225Y08 (226V) isolates revealed 5 amino acid substitutions in the non-structural polyprotein and 4 in the structural polyprotein (Table 4). The A226V substitution was the only variation in the E1 envelope protein and the remaining 3 amino acid substitutions in the structural polyprotein were in E2 envelope protein. At the same time, among the 162 epitopes predicted by the Kolaskar & Tongaonkar algorithm in JEMBOSS (ANTIGENIC) version 1.5 [25], only 2 epitopes coincided with the amino acid differences observed in the structural polyprotein between DS67Y08 (CHIKV-A226) and DS1225Y08 (CHIKV-226V) isolates. Those amino acid substitutions were at residues 677 (E2 envelope protein) and 1035 (A226V in E1 envelope protein) of the structural polyprotein. Though the epitope was similar for both isolates at residue 677, the programme predicted different configurations for the two viral variants at residue 1035 (A226V). CHIKV-226V had a single linear, 34 amino acids long (amino acid positions 1019–1052) epitope, while CHIKV-A226 had 2 short epitopes flanking the same region: a 15 amino acid epitope (amino acid positions 1019–1033) and a 18 amino acid epitope (amino acid positions 1035–1052). Based on these observations, we hypothesized that the structural differences due to the A226V variation may have resulted in 226V antigen being more specific to the paratope of IgM of sera infected with the variant (226V) virus. In the absence of other recognition epitopes, a recombinant E1 antigen with A226 could have much reduced affinity to antibodies produced against the 226V variant, thus rendering a test that relies on an inappropriate A226 *E1* gene ineffective during an outbreak involving CHIKV-226V strain.

**Table 4:** Amino acid differences in structural and nonstructural proteins between D67Y08 (CHIKV-A226) and D1225Y08 (CHIKV-226V).

Polyprotein	Residue	Amino acid in D67Y08 (CHIKV-A226)	Amino acid in D1225Y08 (CHIKV-226V)
Non-Structural polyprotein	67*	A	T
	1074	L	S
	1938*	T	A
	2426	T	I
Structural polyprotein	503	H	R
	577	K	Q
	677*	L	P
	1035* #	A	V

*: residues that are different between the two isolates and overlap with predicted epitopes.

# equivalent to residue 226 in E1 envelope protein; Only the epitope around residue 1035 was predicted to be structurally different between the two isolates.

Using whole virus as antigen (as in the case of MAC-ELISA and EUROIMMUN IFA) offers more antibody recognition sites. As a result, the difference in sensitivity affected by CHIKV-A226 and 226V as antigens was not very prominent. However, as E1 and E2 envelope proteins exist as a heterodimer on the alphavirus surface, contribution of amino acid substitutions in the E2 protein to the observed differences between two antigens could not be underestimated and will be of future interest.

The sensitivity of each RT-PCR protocol was not altered by the virus used. However, the probe-based PCR protocol was at least 10 times more sensitive than the SYBR Green assay. Nevertheless, the SYBR Green method was maintained as the routine test at EHI, due to the following considerations: 1) the cost of the SYBR Green assay was half of that of the probe-based; 2) the SYBR Green assay took 30 minutes, while the probe-based assay required 2.5 hours; and 3) the SYBR Green assay was sensitive enough for routine diagnosis of acute cases. Our previous study has shown that the SYBR Green method was able to detect viral RNA after resolution of fever in 30% of cases [12]. The method also detected three asymptomatic viraemic cases, one day prior to their onset of fever [13]. Taken together, it was concluded that the SYBR method was a cost effective tool for the diagnosis and surveillance of chikungunya fever.

The kinetics of viraemia in patient samples were previously examined and high levels of viraemia were observed during the first 5 days of illness [12]. Combining previous molecular findings [12] and current serology findings, medical practitioners in Singapore have been encouraged to request for PCR-based assays for patients who present within five days of fever and IgM assays for those with fever for more than 5 days. We have found that both EUROIMMUN IFA and MAC-ELISA assays were suitable for outbreaks involving both A226 and 226V variant viruses. For IgM test, MAC-ELISA has the advantage of being cost effective and easy to perform, whereas commercial EUROIMMUN IFA is suitable in laboratories with limited capacity for setting up in-house ELISA systems. An improved rapid test would benefit the community too. For PCR, the SYBR Green protocol was cost effective for the diagnosis of acute patients. This study demonstrates the importance of evaluation of commercial kits and published protocols before application of a diagnostic tool in the clinical and operational settings. With a cost effective and reliable in-house ELISA assay, as demonstrated in this study, the time course of IgM in CHIKV infected individuals is currently being investigated.

## Supporting Information

Checklist S1 STARD checklist.
(0.03 MB DOC)

Data S1 Results of different IgM assays on panel A sera - 8 cases (A1 to A8).
(0.05 MB XLS)

Figure S1 Flow chart for evaluation of CHIK IgM test.
(0.04 MB DOC)
