# Supplementary material for: Evaluation of Chikungunya Diagnostic Assays: Differences in Sensitivity of Serology Assays in Two Independent Outbreaks
Source: PLoS Negl Trop Dis. 2010 Jul 20;4(7):e753. doi: 10.1371/journal.pntd.0000753 (PMC2907414; doi:10.1371/journal.pntd.0000753)
Supplement: Figure S1 — Flow chart for evaluation of CHIK IgM test. (0.04 MB DOC) [file pntd.0000753.s003.doc]

# Supporting Figure S1 - Flow chart for evaluation of CHIK IgM Test

1. **Sensitivity**

CHIKV PCR positive (n= 36) reference test

**Enrollment**

Panel A: 8 patients

n= 28

Consecutive samples collected

Panel B: 28 patients

n= 225

**Evaluation**

CTK

n= 60

EUROIMMUN

n= 60

MAC-ELISA (A226)

n= 60

MAC-ELISA (226V)

n= 60

CTK

n= 74 (10 patients)

MAC-ELISA (A226)

n= 225

EUROIMMUN

n= 225

MAC-ELISA (226V)

n= 225

**Analysis**

- **Average detection threshold** – samples from 18 patients and their subsequent collections were not tested with CTK

(n= 151 )

- **Sensitivity** – only samples collected in first seven days post fever onset were analysed (n= 169)

Excluded from analysis (n= )

Give reasons

- **Average detection threshold** (n= 60 )

- **Sensitivity** – only samples collected in first seven days post fever onset were analysed (n= 41)

1. **Specificity**

Panel C: DENV, JEV, BFV and RRV positive (confirmed by serology, PRNT or characterized by RCPA)

n= 45

CTK

n= 45

all negative

EUROIMMUN

n= 45

all negative

MAC-ELISA (A226)

n= 45

2 positive

43 negative

MAC-ELISA (226V)

n= 45

2 positive

43 negative
